# Supplementary material for: Evaluation of Gremlin-1 as a therapeutic target in metabolic dysfunction-associated steatohepatitis
Source: eLife. 2024 Oct 3;13:RP95185. doi: 10.7554/eLife.95185 (PMC11449483; doi:10.7554/eLife.95185)
Supplement: Supplementary file 1. — (a) Rat choline-deficient, L-amino acid defined high-fat diet (CDAA-HFD) study: clinical chemistry and histological results for all antibody concentrations. (b) Rat CDAA-HFD study: qPCR results for all antibody concentrations. (c) Table of TaqMan assay IDs. (d) Custom-made primer sequences. [file elife-95185-supp1.docx]

**Supplementary Tables**

Evaluation of Gremlin-1 as a therapeutic target in metabolic dysfunction-associated steatohepatitis

Paul Horn, Jenny Norlin, Kasper Almholt, Birgitte M. Viuff, Elisabeth D. Galsgaard, Andreas Hald, Franziska Zosel, Helle Demuth, Svend Poulsen, Peder L. Norby, Morten G. Rasch, Mogens Vyberg, Jan Fleckner, Mikkel Parsberg Werge, Lise Lotte Gluud, Marco R. Rink, Emma Shepherd, Ellie Northall, Patricia F. Lalor, Chris J. Weston, Morten Fog-Tonnesen and Philip N. Newsome

# Supplementary Tables

Supplementary File 1a: Rat CDAA-HFD study: clinical chemistry and histological results for all antibody concentrations

|  | chow | Iso-Ab | 2021 | | | | 0361 | | |
| --- | --- | --- | --- | --- | --- | --- | --- | --- | --- |
|  |  |  | 25 | 2.5 | 1 | 0.25 | 25 | 2.5 | 1 |
| body weight (g) | 639±20 | 459±45 | 443±36 | 438±36 | 442±28 | 447±42 | 473±33 | 453±41 | 446±47 |
| ALT (U/mL) | 72±12 | 197±88 | 170±69 | 247±209 | 155±47 | 194±66 | 149±28 | 170±34 | 191±71 |
| AST (U/mL) | 283±195 | 572±108 | 534±141 | 551±101 | 522±82 | 607±103 | 558±89 | 518±157 | 587±114 |
| relative liver weight (%) | 2.77±0.11 | 5.7±0.79 | 5.72±0.97 | 5.79±1.12 | 6.03±0.86 | 5.45±0.82 | 5.24±0.84 | 5.58±1.01 | 5.48±0.82 |
| steatosis (% area) | 0.85±0.23 | 19.7±2.53 | 17.77±3.41 | 18.46±3.36 | 18.36±3.46 | 16.76±3.01 | 17.78±2.35 | 19.25±2.66 | 18.12±3.54 |
| PSR (% area) | 1.12±0.31 | 5.26±1.61 | 7.11±2.54 | 5.58±2.82 | 6±2.83 | 5.78±2.62 | 4.71±1.03 | 5.54±2.36 | 6±2.59 |
| CD45 (% area) | 1.26±0.67 | 4.89±2.07 | 4.7±1.01 | 4.03±1.14 | 5.87±2.68 | 7.18±2.8 | 4.17±1.35 | 5.81±3.76 | 4.74±2.07 |
| Collagen 1 (% area) | 2.59±1.47 | 10.9±2.6 | 13.62±3.14 | 13.15±6.51 | 13.37±4.44 | 14.35±6.42 | 10.49±2.68 | 11.32±4.14 | 12.62±4.46 |
| aSMA (% area) | 0.83±0.16 | 13.15±3.16 | 13.75±2.41 | 11.82±2.42 | 14±2.67 | 13.56±3.14 | 12.25±2.68 | 12.84±2.05 | 13.43±2.88 |
| CD68 (% area) | 3.79±0.5 | 14.45±2.37 | 15.16±2.11 | 14.81±3.35 | 14.69±2.72 | 15.73±3.27 | 13.34±2.4 | 14.14±2.91 | 14.49±2.51 |
| CD11b (% area) | 0.07±0.04 | 0.68±0.53 | 0.84±0.71 | 0.54±0.42 | 0.91±0.57 | 0.96±0.89 | 0.67±0.5 | 0.9±0.89 | 0.75±0.59 |

All data are given as mean ± SD

Supplementary File 1b: Rat CDAA-HFD study: qPCR results for all antibody concentrations

|  | chow |  | Iso-Ab | 2021 | | | | 0361 | | |
| --- | --- | --- | --- | --- | --- | --- | --- | --- | --- | --- |
|  |  |  |  | 25 | 2.5 | 1 | 0.25 | 25 | 2.5 | 1 |
| Col1a1 | 0±1.13 |  | 6.97±0.78 | 6.95±0.71 | 6.82±0.63 | 7.12±0.95 | 7.39±0.57 | 7.09±0.69 | 7.03±0.73 | 6.97±0.78 |
| Col3a1 | 0±0.72 |  | 3.63±0.68 | 3.4±0.48 | 3.58±0.54 | 3.81±0.58 | 3.97±0.39 | 3.77±0.37 | 3.75±0.52 | 3.79±0.8 |
| Grem1 | 0±0.51 |  | 3.33±0.55 | 3.37±0.37 | 3.28±0.36 | 3.66±0.64 | 3.75±0.46 | 3.49±0.39 | 3.47±0.35 | 3.42±0.35 |
| Mki67 | 0±0.52 |  | 4.53±0.54 | 4.59±0.46 | 4.54±0.69 | 4.83±0.71 | 4.59±0.57 | 4.79±0.54 | 4.55±0.6 | 4.27±0.61 |
| Tgfb1 | 0±1.14 |  | 4.12±1.32 | 4.44±0.62 | 4.24±0.83 | 4.02±2.17 | 4.57±0.53 | 4.08±1.11 | 4.35±0.65 | 4.5±0.64 |
| Timp1 | 0±0.25 |  | 4.35±0.36 | 4.5±0.36 | 4.36±0.51 | 4.5±0.61 | 4.47±0.29 | 4.3±0.4 | 4.26±0.35 | 4.35±0.4 |
| Tnf | 0±1.22 |  | 2.96±1.13 | 3.52±1.09 | 3.1±0.78 | 3.55±0.99 | 3.82±1.11 | 3.54±0.77 | 3.46±0.95 | 3.61±0.91 |

All data are given as mean ± SD

Supplementary File 1c: Table of Taqman Assay IDs

| Species | | Target | | Assay ID |
| --- | --- | --- | --- | --- |
| Human | | ACTA2 | | Hs01879841_s1 |
|  |  | AFTPH | | Hs00214281_m1 |
|  |  | BMP2 | | Hs00154192_m1 |
|  |  | BMP4 | | Hs00370078_m1 |
|  |  | BMP7 | | Hs00233476_m1 |
|  |  | BMPR1A | | Hs04980288_g1 |
|  |  | BMPR1B | | Hs01010965_m1 |
|  |  | BMPR2 | | Hs00176148_m1 |
|  |  | COL1A1 | | Hs00164004_m1 |
|  |  | CTCF | | Hs00902016_m1 |
|  |  | ERCC3 | | Hs01554457_m1 |
|  |  | GREM1 | | Hs01879841_s1 |
|  |  | HPRT1 | | Hs02800695_m1 |
|  |  | ID1 | | Hs00357821_g1 |
|  |  | ID2 | | Hs00747379_m1 |
|  |  | INHBA | | Hs01081598_m1 |
|  |  | INHBB | | Hs00173582_m1 |
|  |  | SMAD1 | | Hs00195432_m1 |
|  |  | SMAD2 | | Hs00998187_m1 |
|  |  | SMAD3 | | Hs00969210_m1 |
|  |  | SMAD4 | | Hs00929647_m1 |
|  |  | SMAD5 | | Hs00195437_m1 |
|  |  | SMAD6 | | Hs00178579_m1 |
|  |  | SMAD7 | | Hs00998193_m1 |
|  |  | SMAD9 | | Hs00931723_m1 |
|  |  | SRSF4 | | Hs00194538_m1 |
|  |  | TIMP1 | | Hs01092512_g1 |
| Rat | | B2m | Rn00560865_m1 |  |
|  |  | Actb | Rn00667869_m1 |  |
|  |  | Gapdh | Rn01775763_g1 |  |
|  |  | Col3a1 | Rn01437681_m1 |  |
|  |  | Col1a1 | Rn01463848_m1 |  |
|  |  | Tgfb1 | Rn00572010_m1 |  |
|  |  | Mki67 | Rn01451446_m1 |  |
|  |  | Timp1 | Rn01430873_g1 |  |
|  |  | TNF | Rn99999017_m1 |  |
|  |  | Grem1 | Rn01509832_m1 |  |

All Taqman assays were purchased from ThermoFisher Scientific

Supplementary File 1d: Custom-made primer sequences

| **Target** | **Primer** | **Sequence** |
| --- | --- | --- |
| GAPDH | GAPDH_116bp_fw | 5' - CTC TGC TCC TCC TGT TCG AC - 3' |
|  | GAPDH_116bp_rv | 5' - CAA TAC GAC CAA ATC CGT TGA C - 3' |
| GFP | GFP_109bp_fw | 5' - GCT ACC CCG ACC ACA TGA AG - 3' |
|  | GFP_109bp_rv | 5' - CGG GTC TTG TAG TTG CCG T - 3' |
| GREM1 | GREM1_109bp_fw | 5' - GAG CCC TGC TTC TCC TCT TG - 3' |
|  | GREM1_109bp_rv | 5' - TCT GAG TCA TTG TGC TGG GC - 3' |
